# Medical Opinion and Movement

**Published:** 1911-07-01

**Authors:** 


### Medical Opinion and Movement.

### Serum Therapy and Urticaria.

The successful treatment of severe cases of herpes gestationis by means of serum injections from a healthy pregnant woman has recently been recorded in these columns. Further confirmation of the efficacy of the treatment is now to hand, and promises to have a yet wider field of application. In the first place serum therapy has been shown to be effective in other forms of toxæmia during pregnancy than skin eruptions. Professor Freund has recorded four such cases. In one the patient's symptoms consisted chiefly of obstinate vomiting, severe neuralgic pains in the extremities, intense jaundice, albuminuria, progressive mental depression and prurigo. The condition completely cleared up after two injections of 25 c.c. of serum from a healthy pregnant woman. The other three patients suffered from puerperal eclampsia, and these also yielded to the treatment, but the serum injections were accompanied by bleeding. Dr. Linser, of Tubingen, also reports several cases of puerperal dermatoses successfully treated by this method, and is so impressed with the efficacy of serum therapy in these cases that he has applied a similar treatment of serum injections from healthy individuals for other skin affections. Several cases of chronic urticaria have been cured in this way, and he further records ten cases of eczema in infants, eight of which were cured after one or two injections, the other two cases proving rather more troublesome. Finally, Dr. Müller, of Tubingen, advises serum injections for atrophic dyspeptic infants affected with enteritis, broncho-pneumonia, or furunculosis, and is of opinion that the serum provides the means of resistance to microbic infections which in ordinary circumstances should be supplied at the maternal breast.

### Syringomyelia.

In the *Deutsche Archiven für Klinische Medizin* Dr. Stursberg gives an account of his observations to determine the nature of the lesion involved in the vasomotor disturbances of syringomyelia, especially whether the centripetal or centrifugal part of the reflex arc is the seat of the lesion. For this purpose he has used the plethysmographic method in five cases. The plethysmograph was applied to one of the arms and registered the result of thermic excitations, especially cold, to one of the other limbs. The application of ice to the other forearm produced a diminution in volume in the arm tested with the plethysmograph, but it was considerably less in amount, less rapid, and of less duration than in a normal individual, whereas on excitation of one of the legs the result was normal in all respects. Dr. Stursberg concludes from this that the vasomotor trouble in syringomyelia depends upon changes in the centripetal portion of the reflex arc, the centrifugal portion being practically normal. It is possible, however, that the loss of the sensation of cold, owing to the thermo-anæsthesia, may to some extent account for the failure to produce the normal reflex effect. The question still remains to be determined whether the centripetal fibres of the vasomotor reflex arc are identical with the thermic sensory fibres. This could only be elucidated by studying a case of syringomyelia in which the affection was strictly unilateral.

### Dr. Brickner's Surgical Suggestions.

In the fourth edition of Dr. W. M. Brickner's little volume of Surgical Suggestions, the number of these practical "wrinkles" has been increased to a thousand. There are very few which will not appeal to British surgeons as accurate or terse statements of sound surgical practice; though it must be admitted that a good many are of the nature of platitudes. Still, there are very many most valuable hints, and few surgeons could read through the book without learning a good deal of useful knowledge. Thus, for instance, in applying atropine solutions to the eye for iritis, it is recommended to press over the inner canthus with the tip of the finger, so as to occlude the tear ducts and thus prevent atropine-laden tears from escaping into the nose, whence they may be swallowed and produce constitutional effects. Then again, a number of most useful warnings are given in connection with the tonsils; suspicion of Hodgkin's disease should be engendered by large, smooth, bilateral tonsillar swellings; status lymphaticus is always to be excluded before suggesting operation under anæsthesia for these cases; herpes of the fauces must be remembered when small reddish spots are seen without surrounding inflammation; and so on. Abdominal palpation in difficult cases is much facilitated by conducting it with the patient in a hot bath—at 105° F.; this secures many of the advantages of examination under an anæsthetic. These are only some of the many aphorisms worth quotation. It is especially noteworthy, as showing the tendency of the day among young surgeons in America, that Dr. Brickner thinks it worth while repeatedly to advise against too hasty resort to operations for many morbid conditions.

### Left Laryngeal Paralysis in Mitral Stenosis.

During the past thirteen years there have been reported thirty-seven cases of mitral stenosis with associated interference with, or abolition of, the movements of the left vocal cord. The explanations advanced to account for this peculiar syndrome have been so varied that Drs. Fetterolf and Norris have investigated the whole of the reported cases. Their conclusions are published in the *American Journal of the Medical Sciences*. They lay stress on the importance of hardened sections for the study of this lesion. It is their conviction, based upon anatomical relations in hardened preparations, that the indirect mechanism may be a variable one; but that when compression accounts for the recurrent paralysis it must always act by squeezing the nerve between the left pulmonary artery and the aorta or the aortic ligament. Anything which dilates or forces upward the left auricle, the left upper pulmonary vein, or the left pulmonary artery will tend to cause the condition, but direct pressure of any portion of a dilated left auricle upon the aortic arch is impossible. When it is remembered that the nerve is normally flattened against the aorta, not rounded, it seems to these observers that its function is abolished not by actual destruction from pressure, but by a neuritis consequent upon a degree of compression which could hardly be sufficient actually to destroy the vitality of the nerve.

### The Treatment of Delirium Tremens.

An exhaustive study of 1,106 cases of delirium tremens treated in two American institutions has been made by Drs. Ranson and Scott. As a result of this extensive experience they do not deal with the plan of hyperalimentation which is still followed by a good many clinicians in this country, confining their remarks to treatment by drugs, chiefly hypnotics and sedatives. Medicinal treatment, they urge, is much more effective in the incipient stages of the disease than later. Attention having been paid to excretion, etc., they advise that incipient cases should receive large doses of the hypnotics, of which veronal is by far the best in their opinion. Whisky should be given regularly, and ergot administered at frequent intervals either by intramuscular injection or by the mouth. This plan of medication should be discontinued gradually, and only after all signs of restlessness and tremor have disappeared. The delirious patient should receive veronal in moderate doses; all other hypnotics, especially morphine and hyoscine, should be withheld from him, but ergot should be given as in the early cases. So far as delirious patients are concerned the data obtained by analysing these 1,106 cases do not afford conclusive evidence whether or not whisky should be regularly employed.

### The Serum Treatment of Hæmophilia.

Evidence is accumulating that in the serum treatment of hæmophilia an agent has at last been discovered which does do good to many cases of that mysterious dyscrasia. The theory upon which the treatment is based is that in hæmophiliacs the normal clotting ferment of the blood (which is either present in the circulating blood or is released during hæmorrhage) is absent, deficient, or held in abeyance. At least it is a fact that human or animal blood serum, applied either locally, subcutaneously, or intravenously, may have a styptic effect during an attack of hæmophilia. In the *Therapeutic Gazette* it is stated that any commercial specific serum may be used in an emergency; but because of the danger of anaphylaxis when alien serum is employed, human serum is the substance of choice. It is also urged that no surgeon should in any circumstances operate upon a hæmophilic person, or on anyone predisposed to bleeding, without resort to a prophylactic injection of serum prior to operation. Subcutaneous administration is as a rule sufficient, and is the preferable method in most cases. For massive hæmorrhages transfusion may also be necessary. The author looks forward to an extension of this treatment to other and much wider fields of usefulness; tuberculosis, eclampsia, pyæmia are only some of the conditions for which he believes it may soon prove efficacious.

### Ether Anæsthesia in Labour.

The comparative disfavour into which chloroform has fallen in the United States has extended even as far as its use in obstetric cases. In this country it has always been the favourite anæsthetic for diminishing the pains of labour, because it affords in small doses such a satisfactory "half-anæsthesia," or, more correctly, an analgesia, without any unpleasant after-effects and without any danger. In the *Journal of the Michigan State Medical Society* Wood condemns the use of chloroform not only for surgical operations but also for this obstetric analgesia; he believes that the objection against ether that either full anæsthesia or none at all can alone be secured to be quite unfounded, and recommends the following practice. At the beginning of each pain five to twenty drops of ether are allowed to fall rapidly upon any simple "open" mask, with the object of enabling the patient to take one or two deep inhalations of fairly strong ether before the pain is well under way. This is said to give a better analgesia than chloroform does, and to obviate also the tendency to post-partum hæmorrhage which chloroform is believed occasionally to engender. The uterine contractions return more promptly than after chloroform, and the anæsthetic may safely be given at an earlier stage if necessary in the case of nervous or neurasthenic patients. There are said to be no ill-effects observed in the child, even after somewhat prolonged administrations.

### Scopolamine-Morphine in Labour.

With the foregoing may usefully be compared and contrasted a critical summary by Dr. J. W. Ballantyne in the *Edinburgh Medical Journal* of the much debated merits of the scopolamine-morphine sequence for inducing the "demi-sommeil" of which the French speak. This method has found few supporters in England, but has been extensively tried in Europe and in Scotland. It is interesting to note that its disadvantages, of which at first but little was ever heard, are gradually attracting attention; Dr. Ballantyne compiles a somewhat formidable list of them. The exact dose is difficult to gauge: a safe dose may not produce the desired effect; an adequate one may introduce risks. After administration the patient must be constantly watched, and should really be in either a hospital or nursing home. Cardiac, pulmonary, and renal lesions are contra-indications. Dryness of the skin and mucous membranes, diminution of glandular secretions, mydriasis, and quickened pulse are merely inconveniences; but delirium, disordered action of the heart, and more or less marked signs of asphyxia are sometimes met with. Unexpected idiosyncrasies are displayed by individual patients to these drugs. Then again the uterine contractions are unfavourably affected, and the action of the abdominal muscles is partially or completely arrested. Lactation and the puerperium are not affected, but nearly all observers report a distinct fœtal mortality. The author concludes that scopolamine-morphine narcosis does not present the profession with an anæsthetic method of labour superior to ether or chloroform inhalations; and this conclusion is reached without invoking the additional argument of the facility with which inhalation can be begun when needed and suspended when the necessity has passed, as contrasted with the fact that when once scopolamine-morphine has been injected, its effect is produced whether or not the labour progresses in the desired manner and at the anticipated rate.

### Puerperal Infection.

In the *Zentralblatt für Gynäkologie*, Dr. Winter, of Königsberg, gives his views in regard to the best method of treatment for cases of abortion with septic infection. As soon as fever develops in a case of abortion a vaginal culture should be taken to determine the nature of the infection. This can easily be done by using an ordinary throat culture-tube, and the bacteriologist can report in twenty-four hours. The culture should be particularly examined for hæmolytic streptococci. If the cultures show complete absence of (or very few) hæmolytic streptococci, in combination with many saprophytes, the uterus may be emptied through a patulous cervix, but if the cervix is rigid he does not advise forcible dilatation. If, on the other hand, pure hæmolytic streptococci are found, all intra-uterine manipulation is strongly contra-indicated. If severe hæmorrhage absolutely necessitates interference, the uterus should be rendered first as free as possible from bacteria by prolonged irrigation and then emptied manually. The curette should not be used in any circumstances. From a study of a large number of cases he is convinced that in those cases in which active interference was used the mortality and complications were increased, and that, on the other hand, no harm resulted from non-interference, even in long-protracted cases of incomplete septic abortion, and in the presence of hæmolytic streptococci delay greatly benefited the patient.

### Cytodiagnosis of Gastric Conditions.

At a recent meeting of the Société Médicale des Hôpitaux, Dr. Loeper read an interesting paper upon the research he had carried out in conjunction with Dr. Binet to determine the nature of the cellular elements contained in the fluid from gastric lavage in different morbid conditions of the stomach. According to these investigations, fairly definite variations in the cellular elements can be made out for the different gastric ailments, and cytology should therefore present a useful means of differential diagnosis in the many dyspeptic conditions of a doubtful nature. In the fasting condition of a normal stomach nothing but cellular, nuclear, and protoplasmic débris is found, without any special characteristics. In gastric ulcer, even in the interval between hæmorrhages, red corpuscles, epithelial cells and leucocytes are found. In cases of gastritis, white corpuscles of every kind are found and glandular elements easily recognisable and often very granular. In cancer of the stomach the fluid from gastric lavage abounds in large cells, deeply stained, very resistant, multi-nucleated, and often rich in glycogen.

### Recurrent Appendicitis.

Professor Volkovitch, of Kiev, draws attention to a new sign which he has discovered in the course of examination of a number of patients presenting themselves for operation on account of recurrent attacks of appendicitis. This sign consists in a change in the muscles of the abdominal wall on the right side, which is evidenced by a flaccid condition of this side of the abdomen. On palpation the abdominal wall appears to be more supple on the right side than on the left, so that the fingers come more easily in contact with the organs in the abdominal cavity, and can take hold of the abdominal wall throughout the entire thickness of it; whereas on the left side the muscular layers of the wall seem to glide under the fingers. The right side of the abdominal wall has lost its elasticity and muscular tone. Professor Volkovitch has observed this condition in about forty cases of recurrent appendicitis examined during the quiescent period, and he concludes that this condition of laxity is due to an atrophic change in the muscles. He points out that there is no contradiction involved in the fact that during the inflammatory process the muscles show an exaggerated degree of tension. On the contrary, the increase in muscular tone during an attack indicates an involvement of the muscular tissues in the inflammatory process, and this being repeated at each recurrent attack leads to nutritional troubles and consequent atrophic condition of the muscles. The author suggests an analogy to the muscular atrophy in the arthropathies as elucidated by Charcot, Paget, and Vulpian. At any rate, whatever may be the pathology, the author thinks it may prove a useful clinical sign.

### Urinary Calculi and Mutton Flesh.

Dr. Lardy, in an article in the *Corresp. Blatt. f. Schweizer Aertz*, attributes to the consumption of mutton flesh the increase in the number of cases of urinary calculi which he has been able to demonstrate in Switzerland. In support of his thesis he states that in Turkey, where he has practised for a number of years, stone is a very common affection, and that the same may be said of England. But both these countries are notorious for a considerable consumption of mutton, and in Switzerland for some years past the importation of this meat, formerly practically nil, has now reached a heavy figure—*e.g.* in 1909 about seven million kilos. The author contents himself with announcing this coincidence and points it out to the attention of research workers.

